# Editorial: Study on plant differentiation between beneficial and pathogenic microorganisms

**DOI:** 10.3389/fpls.2024.1481110

**Published:** 2024-09-10

**Authors:** Jimena Carrillo-Tripp, Sergio de los Santos-Villalobos, Edgardo Sepúlveda, Domingo Martínez-Soto

**Affiliations:** ^1^ Departamento de Microbiología, Centro de Investigación Científica y de Educación Superior de Ensenada (CICESE), Ensenada, BC, Mexico; ^2^ Departamento de Ciencias Agronó micas y Veterinarias, Instituto Tecnológico de Sonora (ITSON), Obregón, Mexico; ^3^ Consejo Nacional de Humanidades, Ciencias y Tecnologías (CONAHCYT) - Departamento de Microbiología, Centro de Investigación Científica y de Educación Superior de Ensenada (CICESE), Ensenada, BC, Mexico

**Keywords:** phytopathogenic microorganisms, beneficial microorganisms, friends and foes, microbial communities, fungi, bacteria, virus

How do plants differentiate friend from foe?. Answering this question is critical to understanding how plants recognize and allow their colonization by beneficial microorganisms, which improve their fitness, adaptation, and survival in hostile environments while deploying a multilayer immune system that thwarts pathogen colonization and avoids diseases (**Figure 1**). The multidisciplinary works published in this Research Topic contribute to answering this burning question in the plant interaction with fungi, bacteria, viruses, and microbial communities.

**Figure 1:**
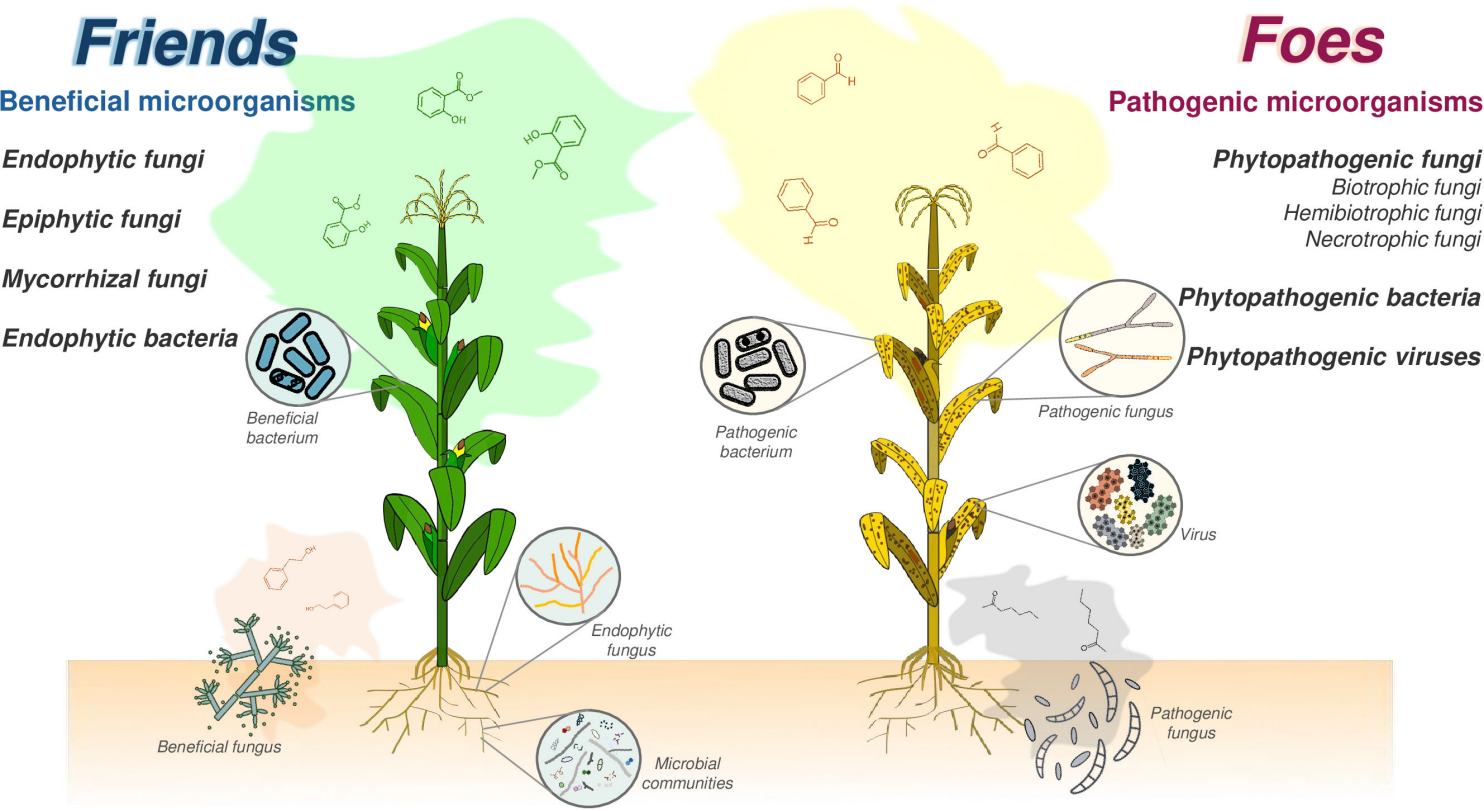
Friends or foes? Graphical representation of beneficial (friends) and detrimental (foes) interactions of plants with microorganisms. The beneficial interactions established by plants with endophytic bacteria, endophytic fungi, mycorrhizae, or microbial communities, improve the plant-priming effect and fitness. Beneficial microorganisms can colonize aboveground tissues, such as stems or leaves, although most beneficial interactions are established belowground with the roots or rhizomes. Detrimental interactions are established between plants and pathogenic bacteria, pathogenic fungi, or viruses. The evident result of these detrimental interactions are disease symptoms shown by the plants as consequences of the attack of the phytopathogen and the plant defense mechanisms. Volatile organic compounds (VOCs) play a key role in the plant-microbe interactions. One of the main questions without a clear response in the plant-microbe interaction is: How do the plants establish interactions with beneficial microorganisms but defy the colonization of pathogens? (reviewed by Harris et al., 2020).

## 1 Fungi-plant interaction

In this Research Topic, López-Coria et al. reported the plant growth promotion and biocontrol activity of *Trichoderma asperellum* against *Fusarium verticillioides*, and the role of this microbial interaction on the expression of the maize diffusional sugar transporters. Thus, in dual confrontation assays, *T. asperellum* showed antagonist activity against the studied phytopathogen, and in the primed plants the infection disease was delayed, and the upregulation of defense-related genes was observed. Additionally, the *T. asperellum* primed plants had longer stems than the non-primed plants. Finally, six maize diffusional sugar transporters increased their expression in the leaves and two at the roots of plants inoculated with *T. asperellum*. On the contrary, *F. verticillioides* downregulated the expression of these transporters on the leaves.

Also related to the fungal-plant interaction, Razo-Belmán et al. presented a review of the fungal volatile organic compounds (FVOCs) as airborne signals perceived by plants, affecting their development, physiology, and priming effect. FOVCs influence the response of plants to microbes. However, despite different plant colonization by beneficial or pathogenic fungi (Martínez-Soto et al., 2023), it is unclear whether plants distinguish VOCs from friends or foes fungi. The authors also discussed five main aspects of FVOCs: *i*) the synthesis of FVOCs, *ii*) sensing of FVOCs by plants, *iii*) plant response to FVOCs from beneficial or pathogenic fungi, *iv*) functions of FVOCs on plants, and *v*) application of FVOCs in agriculture. Being this last point the most important aspect of FVOCs study nowadays.

## 2 Bacteria-plant interaction

Due to their unique microbiome, suppressive soils (SS) prevent plant diseases from developing, even in the presence of pathogens like *Ralstonia solanacearum* and favorable conditions. Meng et al. compared bacterial communities in suppressive and conducive soils for Tobacco bacterial wilt (TBW) and found that SS had higher concentrations of *Bacillus*. They identified *Bacillus velezensis* B4-7, which showed significant suppression of *Ralstonia solanacearum* under acidic conditions. Greenhouse and field trials demonstrated that B4-7 reduced TBW severity, improved tobacco growth, and enhanced plant disease resistance while increasing the beneficial bacterial population in the soil. This work demonstrates how a single bacterial species can contribute to disease suppression through a diverse array of microbial mechanisms, both at the soil and at the plant level.

Similarly, in order to identify beneficial bacteria associated with seeds, by using NGS tools, De-la-Vega-Camarillo et al. present a research work where they identified the bacterial diversity associated with seeds from three Mexican teosinte species: *Zea mays*, *Zea diploperennis*, and *Zea perennis*. The authors identified a core of 38 bacteria related to the teosinte seeds, including *Burkholderia*, *Agrobacterium*, *Erwinia*, *Pseudomonas*, *inter alia*. The bioinformatic analysis of the metabolic pathways in the identified bacteria led the authors to suggest that those bacteria can be involved in plant growth, environmental adaptation, and biological control. Finally, the authors highlight the importance of those growth-promoting bacteria (PGPB) reservoirs from seeds and their putative applications in sustainable agriculture.

## 3 Viruses-plant interaction

In this Research Topic, the mechanisms of how viruses alter metabolic pathways and genetic regulation of their host, changing the cellular environment and resulting in higher viral replication, are explored. Gui et al. compare individual versus mixed infections of two tospoviruses (TSWV and HCRV). Interestingly, gene expression patterns differ in individual infections compared to coinfection. Single infections induce defense and resistance-related proteins. Meanwhile, coinfection does not, a cooperative interaction beneficial to both viruses. Besides showing the predicted function of differential genes and their expected effect on metabolism, the authors studied the expression of several miRNAs. Some are overexpressed during mixed infection and can indirectly turn down the synthesis of defense proteins such as NbPR1, increasing viral susceptibility. The fine-tuned molecular communication between the plant and the virus(es) during the early stage of infection differentiates low- and high-impact viruses.

The effect of viral infection on host metabolism is also thoroughly covered by Jiang et al., who studied how the potyvirus SCMV induced lactate accumulation and anaerobic glycolysis in the plant cells to its advantage, resulting in higher viral titers and more severe symptoms. Overexpressing the enzyme synthesizing lactate, LDH, results in higher viral loads; silencing it reduces virus accumulation. The authors further show how LDH interacts with viral protein 6K2 and how it relocates it to viral replication complexes. Additionally, they unveil how lactate could abolish plant defenses, which correlates with higher viral titer. This work exemplifies how viruses hijack the energy metabolism paths for their own, even when plants could recognize them initially as enemies, as inferred by the production of pathogenesis-related proteins.

## 4 Microbial-plant interactions

Regarding beneficial microorganisms’ consortia improving the plant fitness, Mehdi et al. reported the role of several environmental factors on sucrose accumulation and sugarcane yield, and the negative impact of insufficient water supply, temperature fluctuations, insect pests, and plant diseases. These authors propose reactive oxygen species as a main mechanism to alleviate environmental stresses in sugarcane, as well as the use of endophytic microorganisms as producers of bioactive molecules to enhance plant immune systems and regulate the environmental responses of plants.

On the other hand, it is well-known that Orchidaceae often rely on symbiotic relationships with microorganisms for essential nutrients and overall health. Wang et al. employed high-throughput sequencing to explore the degradation of *Gastrodia elata* (GeB) seedlings across multiple generations of asexual propagation. They observed that the bacterial and fungal composition of GeB seedlings and the surrounding soil changed significantly through each propagation, with a decline in beneficial microbes like *Pseudomonas* and an increase in pathogenic *Penicillium* correlating with disease outbreaks. *Penicillium* antagonistic strains, including *Trichoderma* and *Paenibacillus*, were also isolated from the surrounding soil of propagated GeB, suggesting potential strategies to mitigate degradation.

Altogether, these works shed new light on the mechanisms involved in the plant-microbe interactions and their implications for recognizing friends and resisting foes.
